# Role of Cardiologists in the Management of Systemic Lupus Erythematosus: First Reported Case of Three-vessel Disease in a Young Woman in Pakistan

**DOI:** 10.7759/cureus.5096

**Published:** 2019-07-08

**Authors:** Amara Zafar, Aleena Mohib, Hareem Syed, Sanjay Kumar

**Affiliations:** 1 Medicine, Civil Hospital Karachi, Dow University of Health Sciences, Karachi, PAK; 2 Internal Medicine, Civil Hospital Karachi, Dow University of Health Sciences, Karachi, PAK

**Keywords:** systemic lupus erythematosus (sle), women, coronary artery disease, atherosclerosis, antiphospholipid syndrome

## Abstract

Systemic lupus erythematosus (SLE) is a multi-system autoimmune disorder with predilection towards young women. SLE is associated with increased risk of incidence of cardiac diseases which include atherosclerosis and coronary artery disease (CAD) that can present clinically as angina or myocardial infarction (MI) or be clinically silent in the initial stages. Furthermore, the use of corticosteroids for the treatment of SLE exacerbates the risk of thrombosis, possibly resulting in adverse cardiovascular events in such patients. Antiphospholipid syndrome (APS) is another autoimmune condition which can occur in the setting of SLE, also resulting in hypercoagulability and thrombosis. This goes on to show how important a role cardiologists play in anticipating and managing the cardiac manifestations of SLE, which can significantly reduce the morbidity and mortality of SLE patients. Here we present a case of a young woman with SLE having three-vessel disease, presenting in the cardiac emergency department of Civil Hospital, Karachi with typical chest pain. This case is the first of its kind to be reported in Pakistan, to the best of our knowledge.

## Introduction

Systemic lupus erythematosus (SLE) is a chronic, autoimmune inflammatory disorder that affects multiple organs of the body, most frequently the kidneys and the heart [1]. Because of the multi-system involvement, SLE has a multitude of presentations including ‘butterfly rash’, alopecia, arthralgias, painful breathing, seizures and stroke [2]. However, the strongest association of SLE has been found to be with atherosclerosis and coronary artery disease (CAD), with patients of SLE having seven times greater incidence of CAD than those without SLE [3]. Moreover, women of child-bearing age are the populace most affected by SLE and the incidence of major adverse cardiovascular events like myocardial infarction (MI), is much higher in them than in healthy women [2,4]. Interestingly, the drugs which are used to treat SLE, mainly corticosteroids, are also implicated as risk factors for CAD; thereby, the risk of cardiovascular events in SLE is increased even further [2]. In patients of autoimmune diseases like SLE, cardiovascular events may also be clinically silent in their initial stages [5]. This makes it crucial for these patients to be assessed for a cardiovascular pathology regularly, and the clinical suspicion of such events should always persist even if typical symptoms don’t occur. The antiphospholipid syndrome (APS) is an acquired autoimmune disorder and characterized by hypercoagulability, vascular thrombosis and pregnancy morbidity, with circulating auto-antibodies called antiphospholipid (aPL) antibodies, mainly the lupus anticoagulant (LA) and the anticardiolipin (aCL) antibodies [6]. It is referred to as secondary APS when other autoimmune or inflammatory diseases are present. Cardiac manifestations of APS include valvular disease and Libman-Sacks endocarditis primarily, and also occlusive coronary artery disease which can result in angina and myocardial infarction [6]. Here we present a case of a 32-year-old woman with SLE, being treated with steroids, who subsequently developed cardiac manifestations.

## Case presentation

A 32-year-old married woman, known case of SLE, presented to the cardiac emergency department of a tertiary care hospital in Karachi with the complaint of severe chest pain. The patient was diagnosed with SLE in 2006 when she presented with the complaints of chronic fever of unknown origin, joint pain and oral ulcers. Workup at the time revealed positive anti-double-stranded DNA antibody (anti-dsDNA) with a value of over 1000 IU/ml, positive homogeneous anti-nuclear antibodies (ANA), while both anti-smooth muscle antibodies (ASMA) and anti-mitochondrial antibodies (AMA) were negative. C3 and C4 levels in 2012 were 0.61 (normal 0.9-1.8 g/L) and 0.04 (normal 0.1-0.4 g/L) respectively. She was treated with hydroxychloroquine 400 mg/day and prednisolone 10 mg/day in various combinations with several steroid-free periods for the last 13 years. She was diagnosed with hypertension in 2013 which was managed with losartan 50 mg/day.

In May 2019, she presented to us with severe chest pain for 2 hours. The pain was sudden in onset, diffuse and radiating to the left arm. The pain worsened on exertion and started while she was doing her regular activities in the kitchen. She had no associated nausea, vomiting or shortness of breath. She denied any episodes of chest pain in the past. Her physical examination revealed a blood pressure of 140/90 mmHg, a heart rate of 80 beats/minute, a respiratory rate of 16 cycles/min and temperature of 98 °F. An electrocardiogram (ECG) was obtained which showed a rate of 100 beats per minute (BPM), regular sinus rhythm, left axis deviation, and ST-elevation which was present in Lead 1 and aVL with reciprocal changes (ST-depression) in Lead II, III and aVF, on the basis of which she was diagnosed with high lateral wall MI.

**Figure 1:**
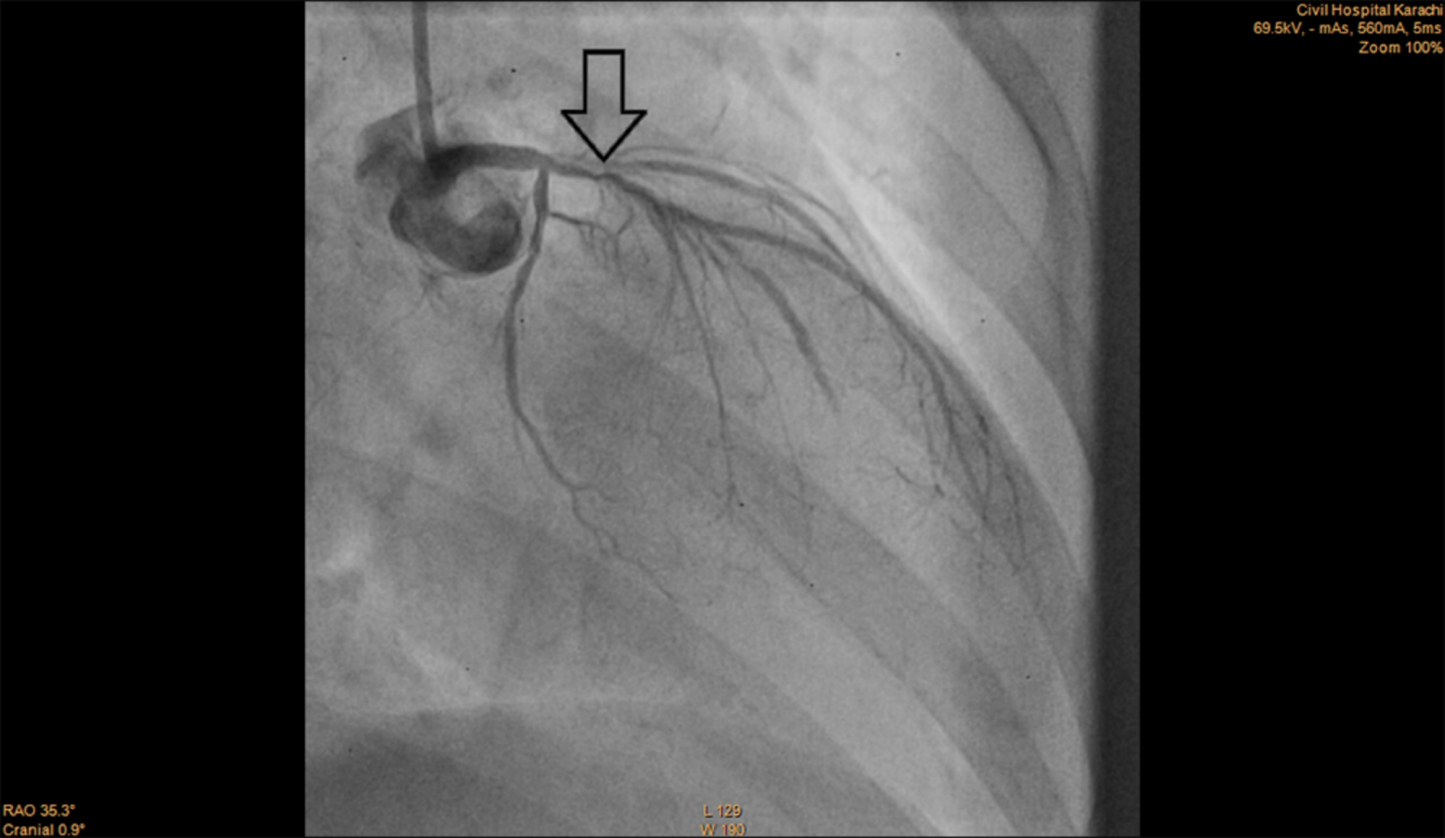
Angiogram of LAD coronary artery showing critical ostial lesion (arrow) LAD: Left anterior descending

**Figure 2:**
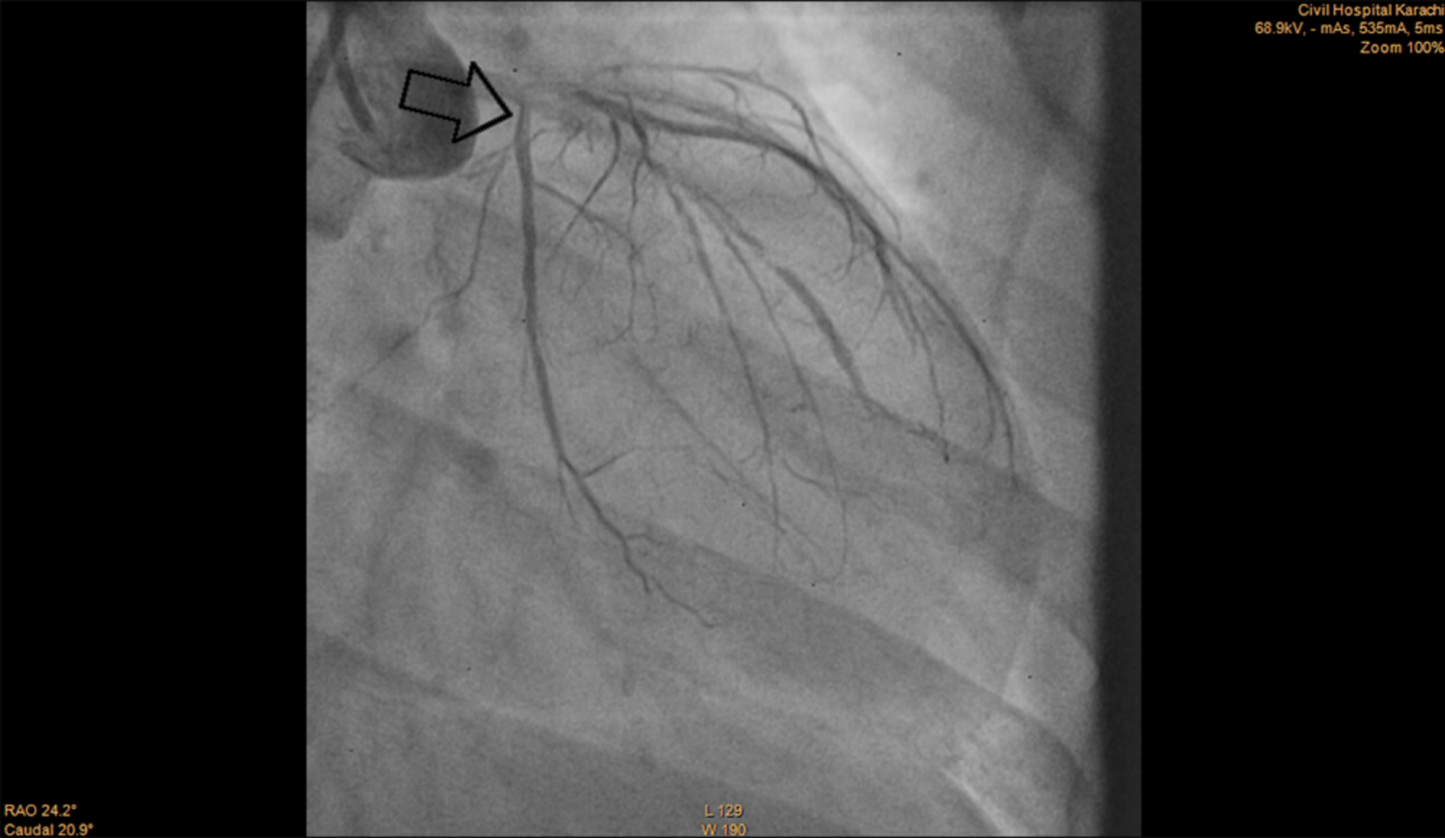
Angiogram of LCx coronary artery showing moderate lesion (arrow) LCx: Left circumflex

**Figure 3:**
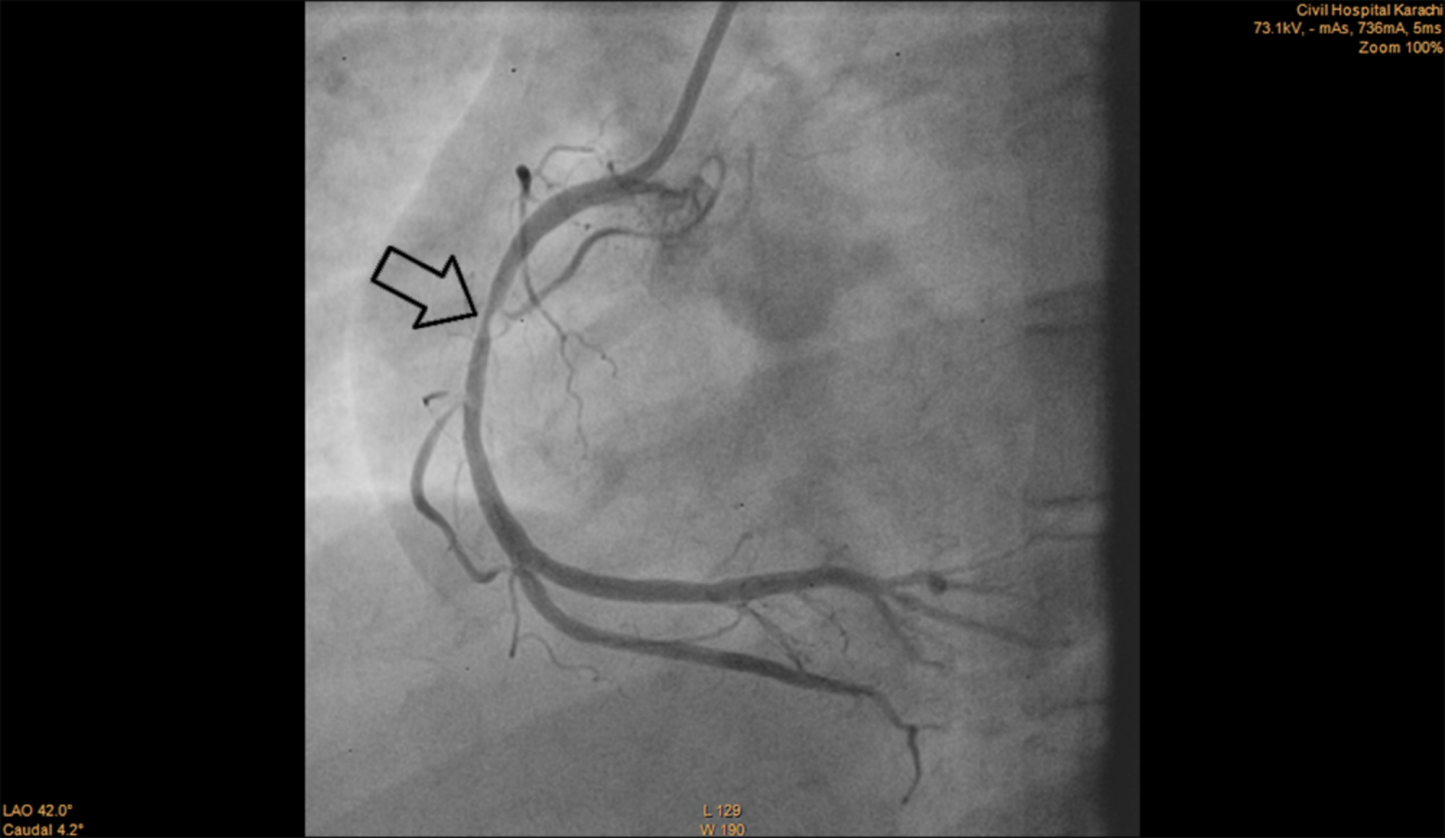
Angiogram of RCA showing severe mid-segment lesion (arrow) RCA: Right coronary artery

Clopidogrel 300 mg and aspirin 300 mg were administered as part of the acute coronary syndrome (ACS) protocol following which she underwent an emergency angiography which revealed severe three-vessel disease. The left main coronary artery (LMCA) had a moderate (40-50%) lesion, left anterior descending (LAD) coronary artery had a critical (80-90%) ostial lesion, ramus intermedius branch had a severe (70-80%) lesion, left circumflex coronary artery (LCx) had a moderate (30-40%) lesion and the right coronary artery (RCA) had a severe (80-90%) mid segment lesion (Figures 1-3). She was kept under observation in the CCU (coronary care unit) and was then transferred to the ICU (intensive care unit) when she was stable.

During admission her complete blood count (CBC), urea, creatinine, electrolytes and liver function tests (LFTs) were within normal limits. Erythrocyte sedimentation rate (ESR) and C-reactive protein (CRP) were elevated at 66 mm/hour (normal 1-20 mm/hour) and 71.7 mg/L (normal 0.0-2.9 mg/L) respectively. Her total protein was found to be 7 g/dL (normal 6-8 g/dL) of which albumin was 2.9 g/dL (normal 3.5-5 g/dL) and globulin was 4.1 g/dL (normal 2-3.5 g/dL). The albumin to globulin (A/G) ratio was found to be 0.71. Her lipid profile was as follows: total cholesterol level was 177 mg/dL (normal < 200 mg/dL), with HDL (high-density lipoproteins), LDL (low-density lipoproteins), and triglycerides levels being 31 mg/dL (normal 35 mg/dL or more), 113 mg/dL (normal < 100 mg/dL) and 164 mg/dL (normal 50-150 mg/dL) respectively. First troponin I level was 0.219 ng/ml (normal < 1.00 ng/ml) while the second one was 1.89 ng/ml. aCL antibodies were negative, while LA was positive with 1.47 giving a possible diagnosis of APS. Echocardiogram showed normal size left ventricle with normal function, no wall motion abnormalities and no valve abnormalities and a normal left ventricular ejection fraction of 60%.

Post-angiography, she was treated with aspirin 300 mg/day, glyceryl trinitrate 2.6 mg twice a day, metoprolol 50 mg/day, atorvastatin 40 mg/day and hydroxychloroquine 200 mg twice a day. Her maintenance dose of steroids was discontinued as she was found to be in remission. The cardio-thoracic surgery team was consulted and a coronary artery bypass grafting (CABG) was planned for the patient, which was successfully performed on the 14th of June 2019. Both left internal mammary artery (LIMA) and saphenous vein graft (SVG) were harvested. However, LIMA showed poor flow of blood after grafting, possibly indicating diffuse vascular disease, hence, SVG was grafted to LAD, ramus intermedius branch and RCA successfully.

## Discussion

The involvement of the heart is one of the major causes of morbidity and mortality in people with SLE, although the overall prognosis of the disease itself has improved. Cardiovascular complications of SLE commonly include pericarditis, myocarditis, endocarditis and CAD [1]. SLE is long known to be one of the strongest risk factors for atherosclerosis and CAD, and studies have shown that the risk of MI is up to nine times greater among SLE patients than the general population, with the risk being 50 times greater in the female patients between the ages of 35 and 44 years [7,8].

Increased total cholesterol levels, obesity, aCL antibodies, long duration of corticosteroid use and previous cardiac involvement with SLE are some risk factors that contribute to the development of CAD in patients with SLE [9-11]. A history of hypertension is also a significant risk factor for the development of CAD in patients with SLE [11]. Although the use of steroids has improved the life expectancy of patients with SLE, this also seems to be causing a rise in the incidence of coronary artery involvement in such patients [12]. This has caused young patients with SLE receiving steroids for their treatment to suffer from angina and MI, which could possibly be due to the formation of atherosclerotic plaques causing stenosis in the coronary vessels that is significant enough to cause clinical symptoms [13,14].

Our patient has been a diagnosed case of SLE since 2006 and was on steroid therapy for the past 13 years. She presented to us with typical chest pain and was eventually diagnosed with high lateral wall MI on ECG. Coronary angiography was done which revealed three-vessel disease. On immunological testing she tested negative for aCL antibodies and positive for LA, giving a possible diagnosis of APS. After consulting the cardio-thoracic surgery team, CABG was planned for the patient and was successfully performed. In 2014, a unique case of a 28-year-old young man with an ST-elevation MI was reported. He presented with a history of chest pain and progressive shortness of breath for the past 24 hours. This man was previously healthy with no cardiovascular risk factors. Immunological tests revealed the presence of antibodies for both SLE and APS and the coronary angiography showed total occlusion of LMCA. He was managed with percutaneous coronary intervention (PCI) which was successful, and he was discharged from the hospital [15]. This suggests that the presence of one or more of these aPL antibodies in SLE patients can be an independent risk factor for accelerated atherosclerosis. In literature we find that aPL antibodies are known to be associated with an increased risk of thrombosis and coronary artery occlusion [16].

Two cases of SLE were reported in 2004, where CABG was performed in women for CAD. The first case was of a 45-year-old woman with SLE for 23 years and receiving steroid treatment for 17 years. She also presented with sub-sternal chest pain, similar to our patient. Coronary angiography revealed 50% narrowing of the LMCA, 70% stenosis in the LAD and 60% stenosis of the circumflex obtuse marginal (OM) 1 artery. CABG was done to LAD and OM 1. The second case was of a 39-year-old woman with SLE, receiving steroids for 17 years whose coronary angiography revealed 80% stenosis in the proximal LAD and CABG was done to LAD [17]. Another case was reported in 2007 of a 42-year-old male hypertensive, diabetic, nonsmoker with SLE. He presented with shortness of breath and class III angina. He was receiving steroid therapy for five years. His coronary angiography was done which revealed a three-vessel disease for which successful CABG was done within two weeks of angiography [18]. These successful cases of CABG surgeries in SLE patients provide corroboration to the fact that a successful CABG has played a potentially life-saving role in our patient as well.

In another case, a 21-year-old female, a diagnosed case of SLE and on steroid therapy for one year presented with chest pain. The cardiac enzymes were normal, and ECG showed ST-segment elevations in leads I, aVL, V1-V6 derivations. Her echocardiography revealed hypokinesia in apical-septal, apical-lateral segments of the heart. Interestingly, her angiography showed normal coronary vessels and both aCL antibodies and LA were negative [19]. In comparison to this, in our patient the echocardiography results were normal, however, the angiography revealed three-vessel disease. Furthermore, LA was also positive in our patient.

Another case of a 51-year-old man presenting with dyspnea was reported in 2014. His ECG showed T-wave inversions in the leads II, III, aVF and V4-V6, and the coronary angiography showed total occlusion of LAD and moderate to severe stenosis in the RCA. His blood tests revealed presence of ANA and anti-dsDNA. He was diagnosed as a case of SLE and stable angina with two-vessel disease. He was managed with an urgent off-pump CABG. Although CABG was successful, the patient deteriorated two days after the surgery, and after further vigorous management for active SLE, he was discharged 60 days later [20]. This shows that CABG in patients with SLE is still a challenge due to multiple organs involved and when emergency surgery is required in a patient presenting with both active SLE and CAD, peri-operative management is a prerequisite and contributes to a successful surgery. Meticulous peri-operative management was done in our patient before CABG was performed which may have been a contributing factor to the success of her surgery. Another factor could be the difference in the age, our patient being a 32-year-old while this man was a 51-year-old.

## Conclusions

With the advancement in the treatment of SLE, the morbidity and mortality associated with SLE have been decreased, but cardiovascular disease is emerging as an important cause of increased mortality in SLE patients. All patients with SLE, therefore, should be screened for the presence of cardiovascular risk factors and the clinicians should have a low threshold for cardiac evaluation in patients with SLE. This case is the first of its kind to be reported in Pakistan, becoming the prototype for future cases of SLE with cardiac manifestations in the region. The successful outcome of this case can have significant implications regarding the role of cardiologists in the anticipation and management of cardiac disease in SLE. Furthermore, it is safe to imply that the possibility of SLE in a young, especially female patient presenting with symptoms corresponding to CAD, should also be considered by the cardiologists. Therefore, this report can be highly instrumental in highlighting the role of cardiologists in the anticipation and management of cases of SLE with cardiac manifestations.
